# Impact of Oncogenic Targets by Tumor-Suppressive *miR-139-5p* and *miR-139-3p* Regulation in Head and Neck Squamous Cell Carcinoma

**DOI:** 10.3390/ijms22189947

**Published:** 2021-09-14

**Authors:** Ayaka Koma, Shunichi Asai, Chikashi Minemura, Sachi Oshima, Takashi Kinoshita, Naoko Kikkawa, Keiichi Koshizuka, Shogo Moriya, Atsushi Kasamatsu, Toyoyuki Hanazawa, Katsuhiro Uzawa, Naohiko Seki

**Affiliations:** 1 Department of Oral Science, Chiba University Graduate School of Medicine, Chiba 260-8670, Japan; axna4812@chiba-u.jp (A.K.); minemura@chiba-u.jp (C.M.); Sachi.o8952@chiba-u.jp (S.O.); kasamatsua@faculty.chiba-u.jp (A.K.); uzawak@faculty.chiba-u.jp (K.U.); 2 Department of Functional Genomics, Chiba University Graduate School of Medicine, Chiba 260-8670, Japan; cada5015@chiba-u.jp; 3 Department of Otorhinolaryngology/Head and Neck Surgery, Chiba University Graduate School of Medicine, Chiba 260-8670, Japan; t.kinoshita@chiba-u.jp (T.K.); naoko-k@hospital.chiba-u.jp (N.K.); kkoshizuka@chiba-u.jp (K.K.); thanazawa@faculty.chiba-u.jp (T.H.); 4 Department of Biochemistry and Genetics, Chiba University Graduate School of Medicine, Chiba 260-8670, Japan; moriya.shogo@chiba-u.jp

**Keywords:** microRNA, expression signature, *miR-139-5p*, *miR-139-3p*, HNSCC, tumor suppressor, passenger strand, *GNA12*, *OLR1*

## Abstract

We newly generated an RNA-sequencing-based microRNA (miRNA) expression signature of head and neck squamous cell carcinoma (HNSCC). Analysis of the signature revealed that both strands of some miRNAs, including *miR-139-5p* (the guide strand) and *miR-139-3p* (the passenger strand) of *miR-139*, were downregulated in HNSCC tissues. Analysis of The Cancer Genome Atlas confirmed the low expression levels of *miR-139* in HNSCC. Ectopic expression of these miRNAs attenuated the characteristics of cancer cell aggressiveness (e.g., cell proliferation, migration, and invasion). Our in silico analyses revealed a total of 28 putative targets regulated by pre-*miR-139* (*miR-139-5p* and *miR-139-3p*) in HNSCC cells. Of these, the *GNA12* (guanine nucleotide-binding protein subunit alpha-12) and *OLR1* (oxidized low-density lipoprotein receptor 1) expression levels were identified as independent factors that predicted patient survival according to multivariate Cox regression analyses (*p* = 0.0018 and *p* = 0.0104, respectively). Direct regulation of *GNA12* and *OLR1* by *miR-139-3p* in HNSCC cells was confirmed through luciferase reporter assays. Moreover, overexpression of *GNA12* and *OLR1* was detected in clinical specimens of HNSCC through immunostaining. The involvement of *miR-139-3p* (the passenger strand) in the oncogenesis of HNSCC is a new concept in cancer biology. Our miRNA-based strategy will increase knowledge on the molecular pathogenesis of HNSCC.

## 1. Introduction

Head and neck squamous cell carcinoma (HNSCC) is a malignant neoplasm that originates in the epithelium of the oral cavity, larynx, and pharynx, and it accounts for approximately 90% of head and neck cancers [1,2]. HNSCC is the sixth most common cancer worldwide, and over 10% of the patients are at an advanced stage at the time of initial diagnosis [3]. In addition, approximately half of the patients will experience recurrence after treatment [4]. Effective treatments for patients with metastasis or recurrence are limited, and the prognosis of patients with treatment failure is extremely poor [5]. The elucidation of the molecular pathogenesis of the malignant transformation of HNSCC is indispensable for the development of new therapeutic strategies.

As a result of the human genome project, a vast number of non-protein-coding RNA molecules (ncRNAs) have been transcribed and have been found to have actual functions in human cells [6,7]. MicroRNAs (miRNAs), a type of ncRNA, are single-stranded RNA molecules consisting of only 19−23 bases [8,9]. A specific characteristic of miRNAs is that a single miRNA can control a large number of RNA transcripts (protein-coding RNAs and ncRNAs) in a sequence-dependent manner [9]. Over the last decade, numerous studies have reported aberrantly expressed (upregulated or downregulated in cancer tissues) miRNAs in a wide range of cancers [10]. Aberrantly expressed miRNAs trigger the disruption of intracellular RNA networks and promote cancer cell initiation, metastasis, and drug resistance [11]. Therefore, up- or downregulated miRNAs in cancer cells are closely involved in human oncogenesis [7].

It is important to determine target miRNAs with altered expression in cancer tissues. Based on this concept, we determined miRNA expression signatures by using RNA sequencing in several cancers [12,13]. Our recent studies showed that some passenger strands (e.g., *miR-145-3p*, *miR-99a-3p*, and *miR-199-3p*) derived from miRNA duplexes act as tumor-suppressive miRNAs by targeting several oncogenes [14,15,16]. According to the previous theory of miRNA biogenesis, miRNA guide strands are incorporated into the RISC to regulate their target genes. In contrast, the passenger strands of miRNAs are degraded in the cytoplasm and have no function [17]. Recent large-scale bioinformatics analyses showed that both strands of several miRNAs (e.g., *miR-30a*, *miR-143*, and *miR-145*) function jointly as tumor-suppressive miRNAs in cancer cells [18,19,20]. The involvement of miRNA passenger strands in human oncogenesis is a new concept in cancer biology.

In this study, we newly generate a miRNA expression signature in clinical specimens of HNSCC by using RNA sequencing. Analysis of the signature is used to reveal the downregulation of *miR-139-5p* (the guide strand) and *miR-139-3p* (the passenger strand) in cancer tissues, and their downregulation is confirmed by an analysis of The Cancer Genome Atlas (TCGA). We demonstrate the tumor-suppressive functions of these two miRNAs and identify their target oncogenes in HNSCC. This is the first report of the involvement of the passenger strand *miR-139-3p* and its target genes in HNSCC. Our miRNA-based strategy will increase knowledge on the molecular pathogenesis of HNSCC.

## 2. Results

### 2.1. Generation of a miRNA Expression Signature in HNSCC

**Figure 1:**
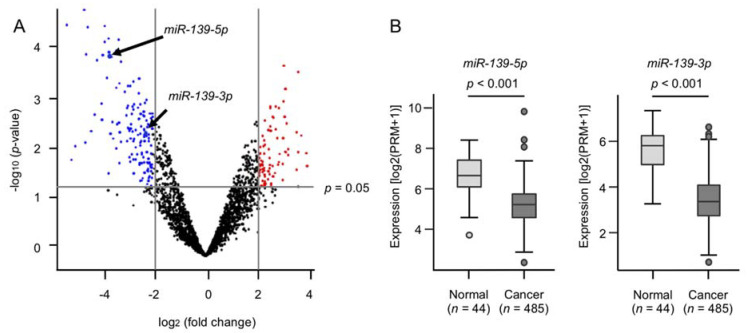
Expression of *miR-139-5p* and *miR-139-3p* in HNSCC tissues. (**A**) Volcano plot of the miRNA expression signature determined through RNA sequencing. The log_2_ fold change (FC) is plotted on the x-axis, and the −log_10_ (*p*-value) is plotted on the y-axis. The blue points represent the downregulated miRNAs with an absolute −log_2_ FC < −2.0 and *p* < 0.05. The red points represent the upregulated miRNAs with an absolute −log_2_ FC > 2.0 and *p* < 0.05. (**B**) The expression levels of *miR-139-5p* and *miR-139-3p* evaluated in an HNSCC dataset from TCGA.

A total of six cDNA libraries were prepared from clinical specimens of HNSCC (three pairs of normal and cancer tissues) and were analyzed by using RNA sequencing. The clinical features of the HNSCC patients are summarized in Table S1. We performed RNA sequencing after a trimming procedure, and 955,314–1,927,436 reads were successfully mapped to miRNA loci within the human genome (Table S2). A total of 112 miRNAs were significantly downregulated (log_2_ fold change < −2.0 and *p*-value < 0.05) in the HNSCC tissues (Figure 1A and Table S3). Notably, our signature showed that both strands of nine pre-miRNAs (*miR-1*, *miR-30a*, *miR-483*, *miR-99a*, *miR-139*, *miR-190a*, *miR-199b*, *miR-204*, and *miR-378a*) were downregulated in the HNSCC tissues (Table S3). We focused on *miR-139* out of these nine pre-miRNAs. To confirm the accuracy of our miRNA signature, we evaluated the expression levels of *miR-139-5p* and *miR-139-3p* in the HNSCC tissues by using the TCGA-HNSC datasets. The expression levels of *miR-139-5p* (*p* < 0.001) and *miR-139-3p* (*p* < 0.001) were significantly lower in the HNSCC tissues (Figure 1B).

### 2.2. Effects of Ectopic Expression of miR-139-5p and miR-139-3p on HNSCC Cell Proliferation, Migration, and Invasion

**Figure 2:**
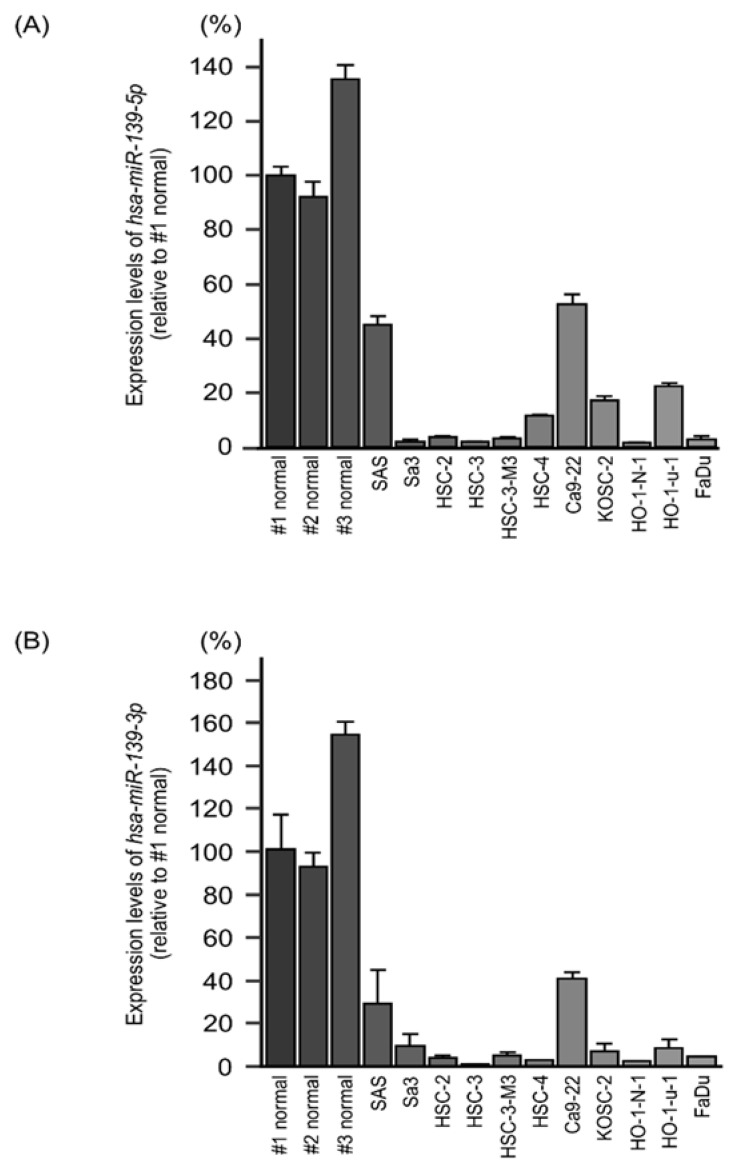
Expression levels of *miR-139-5p* and *miR-139-3p* in the HNSCC cell lines. (**A**,**B**) Expression levels of *miR-139-5p* (**A**) and *miR-139-3p* (**B**) in normal oral tissues and the HNSCC cell lines evaluated with real-time PCR. *RNU48* was used as the internal control. Gene expression levels are expressed relative to those in “#1 normal”.

We examined the expression levels of *miR-139-5p* and *miR-139-3p* and performed functional assays of these miRNAs in two HNSCC cell lines, Sa3 and HSC-3. In these cell lines, the expression levels of *miR-139-5p* and *miR-139-3p* were lower than those in normal epithelial tissues (Figure 2). Normal oral tissues were derived from HNSCC patients, and the details are summarized in Table S1.

**Figure 3:**
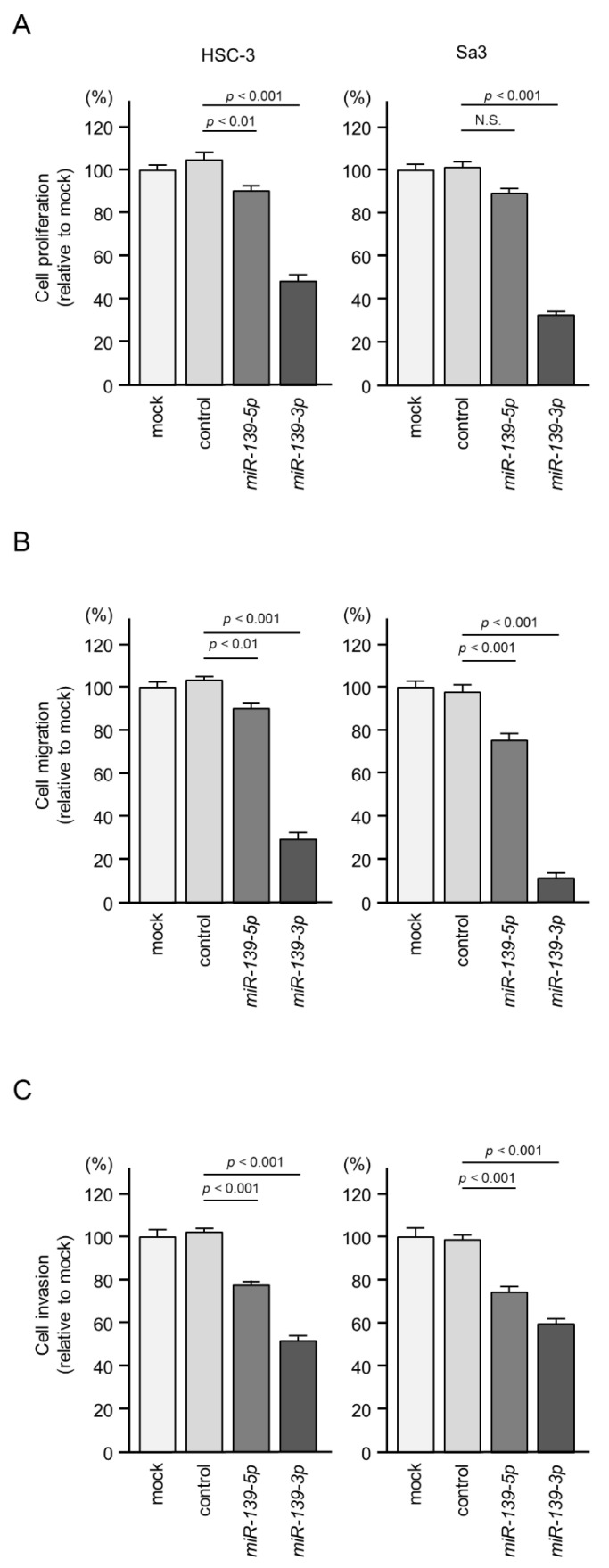
(**A**–**C**) Functional assays of *miR-139-5p* and *miR-139-3p* in HNSCC cells (HSC-3 and Sa3). (**A**) Cell proliferation assessed with an XTT assay at 72 h after transfection of mature miRNAs (N.S.; not significant). (**B**) Cell migration assessed using a membrane culture system at 48 h after seeding miRNA-transfected cells into the chambers. (**C**) Cell invasion determined with a Matrigel invasion assay at 48 h after seeding miRNA-transfected cells into the chambers.

To investigate the tumor-suppressive roles of *miR-139-5p* and *miR-139-3p*, we ectopically expressed mature *miR-139-5p* (UCUACAGUGCACGUGUCUCCAGU) and *miR-139-3p* (UGGAGACGCGGCCCUGUUGGAGU) in Sa3 and HSC-3 cells and assessed the cell functions. Cancer cell malignant features were blocked by the ectopic expression of *miR-139-5p* or *miR-139-3p* (Figure 3A–C). Cell proliferation, migration, and invasion features were markedly suppressed by *miR-139-3p* expression (Figure 3A–C). Typical images of cells from the migration and invasion assays are shown in Figure S1.

### 2.3. Identification of Putative Oncogenic Targets through miR-139-5p and miR-139-3p Regulation in HNSCC Cells

**Figure 4:**
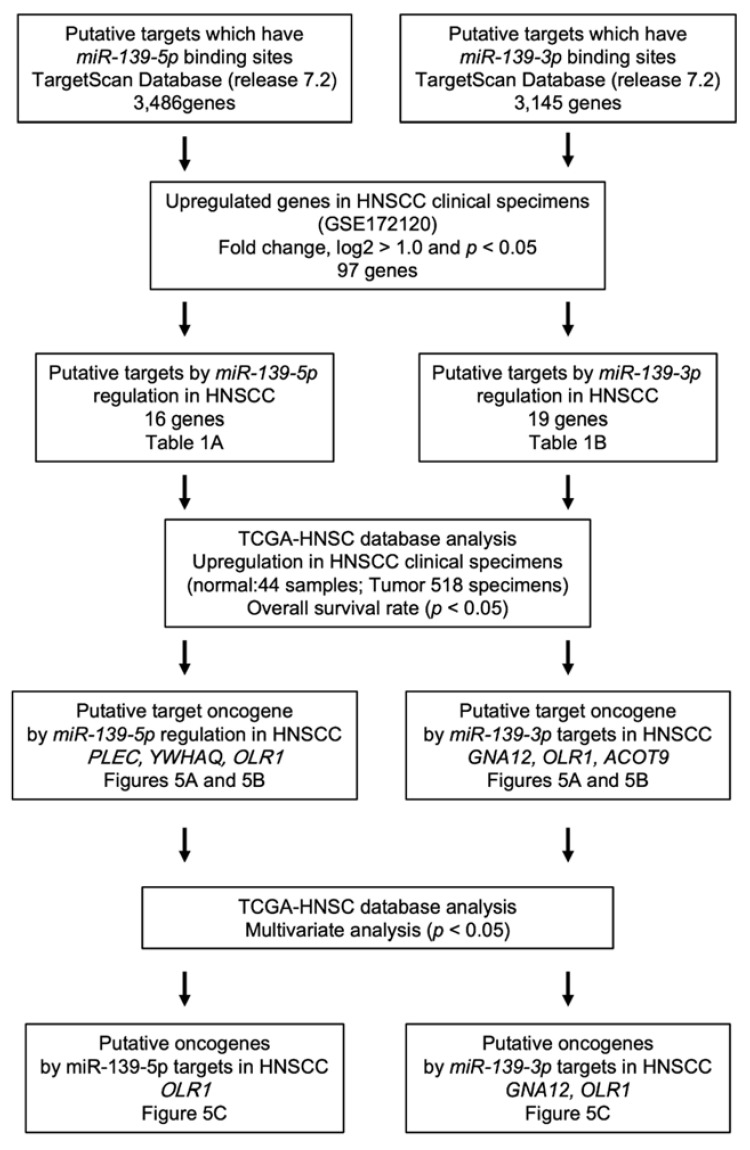
Flowchart of the strategy used to identify the putative oncogenes regulated by *miR-139-5p* and *miR-139-3p* in HNSCC cells.

To identify the putative oncogenic targets of *miR-139-5p* or *miR-139-3p* in HNSCC cells, we used the TargetScanHuman database (release 7.2) and gene expression data (GSE172120: genes upregulated in clinical specimens of HNSCC). Our strategy for identifying the *miR-139-5p* and *miR-139-3p* target genes is shown in Figure 4.

The analysis of the TargetScan database showed that 3486 and 3145 genes had putative *miR-139-5p* and *miR-139-3p* binding sites, respectively, within their sequences (Figure 4). As the tumor suppressor miRNA targets were assumed to be upregulated in cancer tissue, we also performed a genome-wide gene expression analysis in the same clinical specimens used to generate the miRNA signature. The results revealed that 97 genes were significantly upregulated (log_2_ fold change > 1.0 and *p*-value < 0.05) in HNSCC tissues (Table S4).

The TargetScan and gene expression datasets were merged to determine the oncogenic targets of *miR-139-5p* or *miR-139-3p* in HNSCC cells. A total of 28 genes were upregulated in HNSCC tissues, and these genes had a putative *miR-139-5p* or *miR-139-3p* binding site in their 3′UTR (Table 1).

We validated the expression levels of these 28 target genes by using RNA sequencing data obtained from clinical specimens of HNSCC in TCGA. The expression of 24 genes (*BCL11B*, *DOCK5*, *PLEC*, *SRPK2*, *TNFSF10*, *ELAVL2*, *YWHAQ*, *GNA12*, *HIST2H2BF*, *SLC43A2*, *TMEM184B*, *STRIP2*, *PDK1*, *OLR1*, *NAV1*, *MKI67*, *SLC25A37*, *OSBPL3*, *HES6*, *ABCA1*, *ACOT9*, *NKD1*, *APP*, and *SLC16A3*) was upregulated in the HNSCC tissues (*n* = 518) compared with normal tissues (*n* = 44) (Figure S2A,B).

### 2.4. Clinical Significance of the Putative Target Genes of miR-139-5p and miR-139-3p Determined by the TCGA Analysis

**Figure 5:**
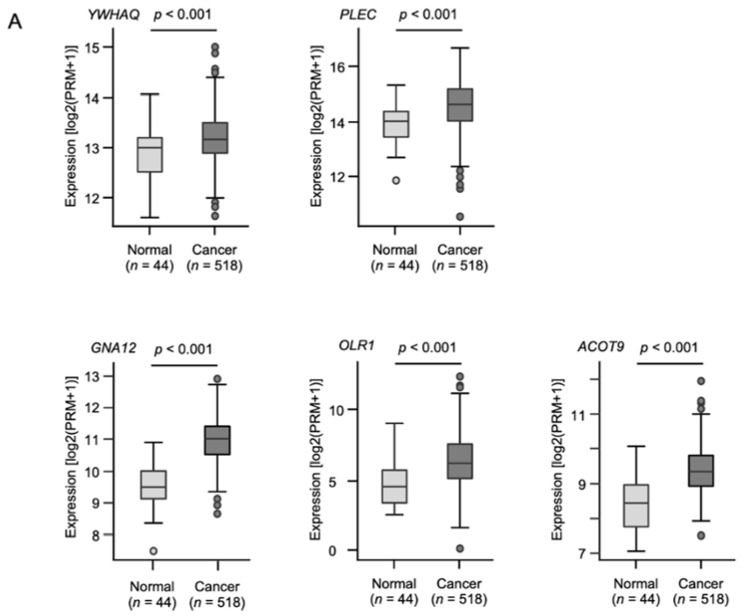

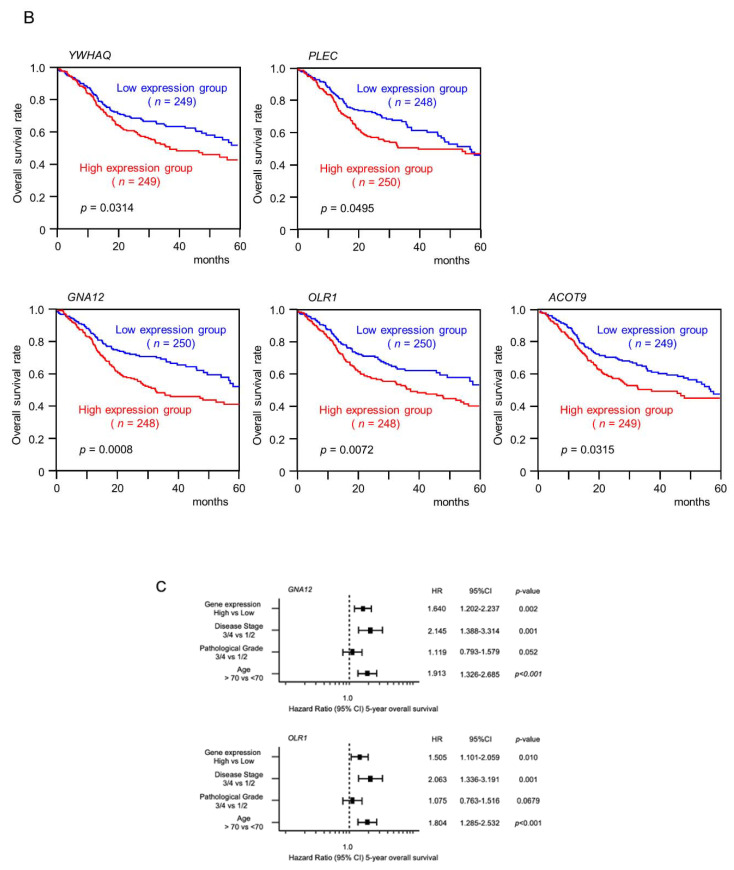
Clinical significance of the expression of putative target genes of *miR-139-5p* and *miR-139-3p* in HNSCC tissues. (**A**) Expression levels of five putative target genes (*PLEC, YWHAQ*, *GNA12*, *OLR1*, and *ACOT9*) in the clinical specimens of HNSCC from a TCGA dataset. All genes were downregulated in HNSCC tissues (*n* = 518) compared with normal tissues (*n* = 44). (**B**) Kaplan–Meier curves of the five-year overall survival rate according to the expression of each gene. Low expression of all five genes was significantly predictive of a worse prognosis in patients with HNSCC. The patients were divided into two groups—the high- and low-expression groups—according to the median miRNA expression level. The red and blue lines represent the high- and low-expression groups, respectively. (**C**) Forest plot presenting the results of a multivariate Cox regression analysis of the prognostic value of two putative target genes (*GNA12* and *OLR1*) identified in an HNSCC dataset from TCGA. The expression levels of *GNA12* and *OLR1* were determined to be independent prognostic factors in terms of the 5-year overall survival rate after adjustments for tumor stage, age, and pathological grade (*p* < 0.05).

To determine clinical usefulness, a clinicopathological analysis of these putative target genes was performed with the TCGA-HNSC data. Five of the genes (*PLEC*, *YWHAQ*, *GNA12*, *OLR1*, and *ACOT9*) were upregulated in HNSCC (Figure 5A), and a significantly worse prognosis was predicted in patients with HNSCC (Figure 5B). Of those genes, *GNA12* and *OLR1* were identified as independent prognostic factors through a multivariate Cox regression analysis (Figure 5C).

### 2.5. Direct Regulation of GNA12 and OLR1 by miR-139-3p in HNSCC Cells

**Figure 6:**
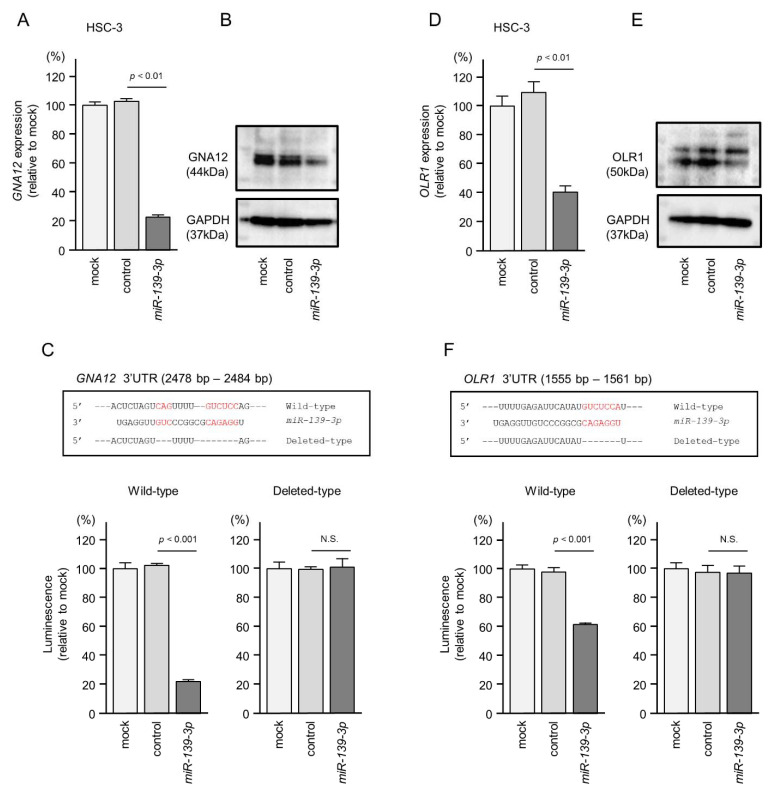
Direct regulation of *GNA12* and *OLR1* expression by *miR-139-3p* in HNSCC cells. (**A**) Real-time PCR showing the significantly reduced expression of *GNA12* mRNA at 72 h after *miR-139-3p* transfection in HSC-3 cells. Expression of *GAPDH* was used as an internal control. (**B**) Western blot showing reduced expression of the GNA12 protein at 72 h after *miR-139-3p* transfection in HSC-3 cells. Expression of GAPDH was used as an internal control. (**C**) One putative *miR-139-3p* binding site predicted within the 3′-UTR of *GNA12* through TargetScanHuman analyses (upper panel). Dual luciferase reporter assays showed reduced luminescence activity after cotransfection of the wild-type *GNA12* 3′-UTR sequence (containing the *miR-139-3p* binding site) with *miR-139-3p* in HSC-3 cells (lower panel). Normalized data were calculated as the Renilla/firefly luciferase activity ratio (N.S., not significant). (**D**,**E**) Real-time PCR and Western blots showing reduced *OLR1* mRNA and protein levels, respectively, after *miR-139-3p* transfection in HSC-3 cells. (**F**) One putative *miR-139-3p* binding site predicted within the 3′-UTR of *OLR1* through TargetScanHuman analyses (upper panel). Luminescence activity was reduced after cotransfection of the wild-type *OLR1* 3′-UTR sequence (containing the *miR-139-3p* binding site) with *miR-139-3p* in HSC-3 cells.

To investigate whether the expression of *GNA12* and *OLR1* is regulated directly by pre-*miR-139* in HNSCC cells, we performed real-time PCR, Western blotting, and dual luciferase reporter assays. In HSC-3 cells transfected with *miR-139-3p*, both the mRNA and protein levels of *GNA12* were markedly reduced (Figure 6A,B).

The dual luciferase reporter assays showed that *miR-139-3p* binds directly to *GNA12*. Luciferase activity was significantly reduced after transfection of a vector harboring the wild-type *GNA12* 3′-UTR sequence (containing the *miR-139-3p* binding site). In contrast, luciferase activity was not reduced after transfection of a vector harboring a *GNA12*-deleted sequence (lacking the *miR-139-3p* binding site) (Figure 6C). These data suggest that *miR-139-3p* binds directly to the 3′-UTR of *GNA12* to control its expression.

The TargetScan database shows that the binding sites of both *miR-139-5p* and *miR-139-3p* are located within the 3′UTR of the *OLR1* gene. We investigated the involvement of these miRNAs in the expression control of *OLR1* in HNSCC cells. As a result, *miR-139-5p* was not involved in *OLR1* expression (Figure S3).

Both the mRNA and protein levels of *OLR1* were significantly downregulated after *miR-139-3p* transfection in HSC-3 cells (Figure 6D,E). Luciferase activity was significantly reduced after transfection with a vector harboring the wild-type sequence—but not with that harboring a deleted sequence—of the *OLR1* 3′-UTR (Figure 6F). These data suggest that *miR-139-3p* directly controls *OLR1* expression by binding to its 3′-UTR in HNSCC cells.

### 2.6. Overexpression of GNA12 and OLR1 in Clinical Specimens of HNSCC

**Figure 7:**
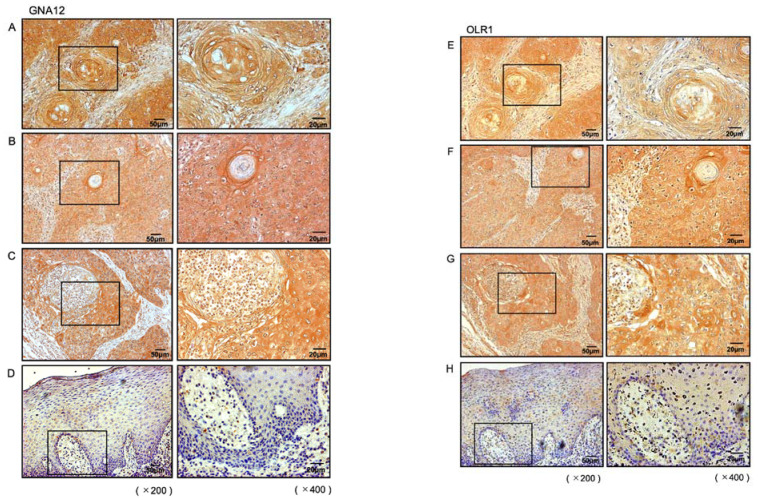
Overexpression of GNA12 and OLR1 in clinical specimens of HNSCC. (**A**–**D**) Immunohistochemical staining of GNA12 in clinical specimens of HNSCC. High expression of GNA12 was detected in the nuclei and/or cytoplasm of cancer cells (**A**–**C**), and weak expression was detected in normal oral mucosa (**D**). (**E**–**H**) Immunohistochemical staining of OLR1 in clinical specimens of HNSCC. High expression of OLR1 was detected in the nuclei and/or cytoplasm of cancer cells (**E**–**G**), and weak expression was detected in normal oral mucosa (**H**).

The expression of the GNA12 and OLR1 proteins was evaluated by immunostaining in clinical specimens of HNSCC. High expression of GNA12 was detected in HNSCC lesions (Figure 7A–C). In contrast, normal epithelial tissues showed weak immunostaining (Figure 7D). High expression of OLR1 was detected in HNSCC lesions, in contrast to the weak staining in the normal epithelium (Figure 7E–H). The clinical features of three of the HNSCC cases used for immunohistochemical staining are summarized in Table S5.

### 2.7. GNA12- and OLR1-Mediated Pathways in HNSCC Cells

Finally, we investigated the genes that were differentially expressed between the high and low *GNA12* or *OLR1* expression groups in an HNSCC cohort from TCGA using a gene set enrichment analysis. As a result, 19 and 14 gene sets were significantly enriched (FDR *q*-value < 0.05) in the high *GNA12* and *OLR1* expression groups, respectively (Table 2). The most enriched gene set in both the *GNA12* and *OLR1* expression groups was that of the “epithelial–mesenchymal transition” (Table 2 and Figure S4). These results indicate that *GNA12* and *OLR1* play roles as oncogenes in HNSCC via the epithelial–mesenchymal transition pathway.

## 3. Discussion

Due to the high rate of recurrence and metastasis after the initial treatment, the prognosis of patients with HNSCC remains poor [4,21]. Recently developed molecule-targeted drugs and immune checkpoint inhibitors have not significantly improved the prognosis of patients [5,22,23]. Therefore, novel treatment options for patients with advanced HNSCC are urgently needed. The elucidation of the molecular mechanisms underlying the oncogenesis of HNSCC is indispensable for finding therapeutic target molecules.

We have continued to use miRNA-based approaches in order to identify potential prognostic markers and therapeutic targets for HNSCC [24]. Our previous studies showed that certain integrins are controlled directly by tumor-suppressive miRNAs (e.g., *miR-29*-family, *miR-150*, and *miR-199a*) and that the activation of signaling mediated by *ITGA3/ITGB1* and *ITGA6* enhances the aggressiveness of HNSCC [12,16,25]. In addition, the expression of *ITGA3* predicted poorer survival of patients with HNSCC [16].

Recently available small-RNA-sequencing methods are suitable for generating miRNA expression signatures in a wide range of human cancers. Our research group has generated miRNA signatures in several types of cancers [13,26,27,28,29]. Interestingly, these signatures revealed that some passenger strands of miRNAs (e.g., *miR-99a-3p*, *miR-101-3p*, *miR-144-5p*, *miR-145-3p*, and *miR-150-3p*) are downregulated in cancer tissues, and these miRNAs actually function as tumor suppressors [15,27,29,30,31]. The original miRNA theory indicates that the passenger strand of a miRNA duplex is degraded in the cytoplasm and is nonfunctional [17]. Recently, a large-scale in silico analysis of miRNAs in more than 5200 patient samples representing 14 different cancers was performed. That study demonstrated that some miRNAs (e.g., both strands of *miR-28*, *miR-30a*, *miR-139*, *miR-144*, and *miR-145*) coordinately modulate oncogenic pathways [32]. Simultaneous analysis of miRNA-controlled oncogenic networks will lead to the elucidation of new molecular mechanisms in human cancers.

In this study, we determined the miRNA expression signature of HNSCC by using clinical specimens. The analysis of the signature showed that a total of 112 miRNAs were significantly downregulated (log_2_ fold change < −2.0 and *p*-value < 0.05) in cancer tissues. Interestingly, among these downregulated miRNAs, 31 were annotated as passenger strands of miRNAs in the miRBase database. Functional analysis of these miRNAs and their regulated molecular networks will help elucidate the molecular pathogenesis of HNSCC.

Our previous studies revealed that *miR-139-3p* (the passenger strand of pre-*miR-139*) acts as a tumor-suppressive miRNA in bladder cancer [33] and renal cell carcinoma [34]. A single miRNA can control a vast number of RNAs in cells, and the genes controlled by a miRNA vary from cell to cell. In this study, we revealed that both strands of pre-*miR-139* act as tumor-suppressive miRNAs in HNSCC cells. Previously, a vast number of studies reported that *miR-139-5p* plays a tumor-suppressive role in multiple cancers [35]. In oral squamous cell carcinoma (OSCC), *miR-139-5p* inhibited tumorigenesis by targeting *HOXA9* and *CXCR4* [36,37]. Recent studies showed that long non-coding RNAs (lncRNAs) are closely associated with human oncogenesis [11]. Several studies showed that *LINC00152* acts as a cancer-promoting and metastatic lncRNA in multiple cancers [38]. A recent study indicated that the lncRNA *LINC00152* adsorbs *miR-139-5p* and behaves as an oncogene in OSCC [39].

On the other hand, because it is a passenger strand, few studies have evaluated the functional significance of *miR-139-3p* in HNSCC cells. A previous study showed that the expression of *miR-139-3p* was suppressed by human papillomavirus 16, which, in turn, promoted oncogenesis in head, neck, and cervical cancers [40]. Another study showed that *miR-139-3p* is significantly downregulated in laryngeal squamous cell carcinoma and that its expression regulates several cancer-related genes, (e.g., *RAB5A*, *ITGB1*, *FAK*, *PXN*, *VEGF*, and *MMP9*) [41]. In breast cancer, expression of *miR-139-3p* was downregulated in cancer tissues and cell lines, and its expression was associated with a poor prognosis in breast cancer patients [42]. Overexpression of *miR-139-3p* inhibited malignant cancer cell phenotypes by targeting *RAB1A* [42]. Judging from previous studies and our studies, both the guide and passenger strands of *miR-139* have tumor-suppressive functions, and the search for molecular pathways controlled by each strand will contribute to the elucidation of the molecular mechanisms of HNSCC.

Our next focus was to identify the oncogenic targets regulated by each strand of *miR-139* (*miR-139-5p* and *miR-139-3p*) in HNSCC cells. Our in silico analysis identified five genes (*PLEC*, *YWHAQ*, *GNA12*, *OLR1,* and *ACOT9*) that were closely associated with a poor prognosis of patients with HNSCC. Importantly, two of those genes (*GNA12* and *OLR1*) were independent prognostic markers in patients with HNSCC.

*GNA12* (*Ga12*) is the alpha subunit of G proteins (heterotrimeric guanine nucleotide-binding protein), which are classified into four subfamilies: Gs, Gi, Gq, and G12 [43]. *Ga12* and RhoGEF (Rho guanine nucleotide exchange factors) activate Rho-mediated signaling, which, in turn, enhances cancer cell progression, invasion, and metastasis [44]. High expression of *GNA12* was detected in OSCC patients and contributed to cancer cell migration and invasion and lymph node metastasis [45,46].

A previous study showed that *OLR1* is a lectin-like scavenger receptor. This receptor binds to several ligands, such as oxidized low-density lipoprotein, polyanionic chemicals, and anionic phospholipids [47]. Aberrant expression of *OLR1* has been reported in multiple cancers; its expression induces the release of inflammatory cytokines and is closely related to cancer development and metastasis [48]. Detailed functional analyses of these genes will provide important information for elucidating the molecular pathogenesis of HNSCC.

In conclusion, our new RNA-sequencing-based miRNA signature revealed aberrantly expressed miRNAs in HNSCC tissues, including certain passenger strands of miRNAs. Both strands of pre-*miR-139* (*miR-139-5p* and *miR-139-3p*) acted as tumor-suppressive miRNAs in HNSCC cells. A total of 28 genes were identified as targets of these miRNAs. Among these targets, *GNA12* and *OLR1* were demonstrated to be independent prognostic markers in patients with HNSCC. Identification of novel tumor-suppressive miRNAs and their regulated oncogenic targets may improve our knowledge of the molecular mechanisms of the oncogenesis of HNSCC.

## 4. Materials and Methods

### 4.1. Clinical HNSCC Specimens, Normal Epithelial Specimens, and HNSCC Cell Lines

Total RNA was extracted from three paired HNSCC and three normal oral epithelial tissue specimens for RNA sequencing and microarray analysis. The clinical features of HNSCC patients are summarized in Table S1. This study was approved by the Bioethics Committee of Chiba University (approval number: 28–65, 10 February 2015). Informed consent was obtained from all patients. The study’s methodologies conformed to the standards set by the Declaration of Helsinki.

The HNSCC cell lines used in this study were obtained from the RIKEN BioResource Center (Tsukuba, Ibaraki, Japan) and are summarized in Table S6.

### 4.2. Generation of a miRNA Expression Signature in HNSCC through RNA Sequencing

To construct the miRNA expression signature of HNSCC, six cDNA libraries were sequenced by using HiSeq 2000 (Illumina, San Diego, CA, USA). The small-RNA-sequencing and data-mining procedures have been described in our previous studies [12,26,27,28].

### 4.3. Ectopic miRNA Expression Assays of Cell Proliferation, Migration, and Invasion

The procedures used for transient transfection assays of miRNAs, siRNAs, and plasmid vectors were described in our previous studies [12,14,15,16]. The reagents used are listed in Table S7.

### 4.4. Identification of Putative Targets Controlled by miR-139-5p and miR-139-3p in HNSCC Cells

The strategy used for the selection of the *miR-139-5p* and *miR-139-3p* target genes in this study is summarized in Figure 4. We selected putative target genes with the *miR-139-5p* and *miR-139-3p* binding sites by using TargetScanHuman ver. 7.2 (http://www.targetscan.org/vert_72/, accessed on 10 July 2020) [49]. The expression profiles of the clinical specimens of HNSCC (genes downregulated in HNSCC tissues) were used to screen miRNA target genes. Our expression data were deposited in the GEO database (accession number: GSE172120). Furthermore, we narrowed down the candidate genes by factoring in clinical information from TCGA-HNSC analyses.

For the Kaplan–Meier survival analysis, we downloaded TCGA-HNSC clinical data (TCGA, Firehose Legacy) from cBioportal (https://www.cbioportal.org, accessed 10 April 2020). Gene expression data for each gene were collected from OncoLnc (http://www.oncolnc.org, accessed on 20 April 2021) [50]. For the log-rank test, we used JMP Pro 15 (SAS Institute Inc., Cary, NC, USA).

Multivariate Cox regression analyses were also performed with the TCGA-HNSC clinical data and survival data according to the expression levels of each gene from OncoLnc to identify factors associated with HNSCC patient survival. In addition to gene expression, the tumor stage, pathological grade, and age were evaluated as potential independent prognostic factors. The multivariate analyses were performed using JMP Pro 15.0.0 (SAS Institute Inc., Cary, NC, USA).

### 4.5. Direct Regulation of Target Genes by miR-139-3p Using Dual Luciferase Reporter Assays

The psiCHECK-2 vector (C8021; Promega, Madison, WI, USA) was inserted into the synthesized DNA with or without the *miR-139-3p* binding sequences of *GNA12* or *OLR1.* The plasmid vectors were then transfected into cells using Lipofectamine 2000 (Invitrogen) at a final concentration of 50 ng/well.

### 4.6. Western Blotting and Immunohistochemistry

The Western blotting and immunohistochemistry procedures were detailed in our previous study [12]. The antibodies used are listed in Table S7. The clinical features of 3 HNSCC cases used for immunohistochemical staining are summarized in Table S5.

### 4.7. Analysis of Molecular Pathways Related to Target Genes Controlled by miR-139-3p

To analyze the molecular pathways related to the target genes of *miR-139-3p*, we performed gene set enrichment analysis (GSEA) for *GNA12* and *OLR1*.

We divided the TCGA-HNSC RNA-seq data into a high-expression group and low-expression group according to the Z-score of *GNA12* or *OLR1* on cBioportal. A ranked list of genes was generated by comparing the expression levels of each gene between the two groups. We loaded the obtained gene list into gene set enrichment analysis (GSEA) software [51,52] and used the Molecular Signatures Database (MSigDB) hallmark gene set [53].

### 4.8. Statistical Analysis

All data were presented as mean values and standard errors of at least three independent experiments. Statistical analyses were performed using JMP Pro 15 (SAS Institute Inc., Cary, NC, USA). Differences between 2 groups were evaluated with t-tests. Dunnet’s test was used for multiple-group comparisons. *p*-value < 0.05 was considered statistically significant.

## 5. Conclusions

In this study, we focused on *miR-139-5p* and *miR-139-3p* based on our miRNA signatures. Our functional assays indicated that these miRNAs play an oncogenic role in HNSCC cells. Using an in silico database analysis to identify the gene targets regulated by *miR-139-5p* and *miR-139-3p*, we rapidly identified candidate oncogenes in HNSCC. Our HNSCC miRNA signature and miRNA-based analyses will provide important insights into the molecular pathogenesis of HNSCC.

## Figures and Tables

**Table 1:** Candidate target genes regulated by *miR-139-5p* and *miR-139-3p.*

**A. Sixteen Candidate Target Genes Regulated by *miR-139-5p***					
**Entrez** **Gene ID**	**Gene Symbol**	**Gene Name**	**Fold Change** **(log_2_ > 1.0)**	***p*** **-Value**	**Total** **Sites**
19	*ABCA1*	ATP-binding cassette, sub-family A (ABC1), member 1	2.065	0.043	1
64919	*BCL11B*	BAF chromatin remodeling complex subunit BCL11B	2.620	0.030	0
80005	*DOCK5*	dedicator of cytokinesis 5	1.940	0.040	0
1993	*ELAVL2*	ELAV-like neuron-specific RNA-binding protein 2	2.606	0.013	2
4973	*OLR1*	oxidized low-density lipoprotein (lectin-like) receptor 1	4.620	0.035	1
26031	*OSBPL3*	oxysterol-binding protein-like 3	1.799	0.038	1
5163	*PDK1*	pyruvate dehydrogenase kinase, isozyme 1	1.270	0.015	1
5209	*PFKFB3*	6-phosphofructo-2-kinase/fructose-2,6-biphosphatase 3	1.840	0.040	0
5339	*PLEC*	plectin	1.020	0.040	0
10154	*PLXNC1*	plexin C1	1.463	0.038	1
51312	*SLC25A37*	solute carrier family 25 (mitochondrial iron transporter), member 37	1.740	0.040	0
6733	*SRPK2*	SRSF protein kinase 2	1.550	0.050	0
91937	*TIMD4*	T cell immunoglobulin and mucin domain containing 4	2.140	0.010	0
25829	*TMEM184B*	transmembrane protein 184B	1.120	0.046	2
8743	*TNFSF10*	TNF superfamily member 10	3.540	0.040	0
10971	*YWHAQ*	tyrosine 3-monooxygenase/tryptophan 5-monooxygenase activation protein, theta	1.059	0.047	1
**B. Nineteen Candidate Target Genes Regulated by *miR-139-3p***					
**Entrez** **Gene ID**	**Gene Symbol**	**Gene Name**	**Fold Change** **(log_2_ > 1.0)**	***p*** **-value**	**Total** **sites**
2768	*GNA12*	guanine nucleotide-binding protein (G protein) alpha 12	1.114	0.039	1
440689	*HIST2H2BF*	histone cluster 2, H2bf	2.816	0.035	2
124935	*SLC43A2*	solute carrier family 43 (amino acid system L transporter), member 2	1.962	0.045	1
25829	*TMEM184B*	transmembrane protein 184B	1.120	0.046	2
57464	*STRIP2*	striatin interacting protein 2	2.572	0.047	1
5163	*PDK1*	pyruvate dehydrogenase kinase, isozyme 1	1.270	0.015	1
10154	*PLXNC1*	plexin C1	1.463	0.038	1
4973	*OLR1*	oxidized low-density lipoprotein (lectin-like) receptor 1	4.620	0.035	1
55784	*MCTP2*	multiple C2 domains, transmembrane 2	1.274	0.035	2
89796	*NAV1*	neuron navigator 1	1.998	0.036	1
4288	*MKI67*	marker of proliferation Ki-67	1.005	0.048	1
51312	*SLC25A37*	solute carrier family 25 (mitochondrial iron transporter), member 37	1.739	0.044	1
26031	*OSBPL3*	oxysterol-binding protein-like 3	1.799	0.038	1
55502	*HES6*	hes family bHLH transcription factor 6	1.782	0.048	1
19	*ABCA1*	ATP-binding cassette, sub-family A (ABC1), member 1	2.065	0.043	1
23597	*ACOT9*	acyl-CoA thioesterase 9	1.121	0.034	1
85407	*NKD1*	naked cuticle homolog 1 (Drosophila)	1.215	0.042	1
351	*APP*	amyloid beta (A4) precursor protein	1.412	0.046	1
9123	*SLC16A3*	solute carrier family 16 (monocarboxylate transporter), member 3	2.763	0.046	1

**Table 2:** The significantly enriched gene sets.

**A. Gene Sets in the High *GNA12* Expression Group**		
**Name**	**Normalized Enrichment Score**	**FDR *q*-Value**
epithelial–mesenchymal transition	3.685	*q* < 0.001
myogenesis	3.268	*q* < 0.001
UV response dn	2.605	*q* < 0.001
angiogenesis	2.561	*q* < 0.001
KRAS signaling up	2.543	*q* < 0.001
inflammatory response	2.434	*q* < 0.001
apical junction	2.411	*q* < 0.001
coagulation	2.407	*q* < 0.001
TNF-alpha signaling via NFkb	2.309	*q* < 0.001
hypoxia	2.119	*q* < 0.001
TGF-beta signaling	2.096	*q* < 0.001
IL6/JAK/STAT3 signaling	2.059	*q* < 0.001
hedgehog signaling	2.005	*q* < 0.001
complement	1.891	0.001
notch signaling	1.788	0.004
IL2/STAT5 signaling	1.772	0.004
pancreas beta cells	1.645	0.012
mitotic spindle	1.554	0.028
apoptosis	1.506	0.040
**B. Gene Sets in the High *OLR1* Expression Group**		
**Name**	**Normalized Enrichment Score**	**FDR *q*-Value**
epithelial–mesenchymal transition	2.309	*q* < 0.001
inflammatory response	2.152	*q* < 0.001
myogenesis	2.121	*q* < 0.001
angiogenesis	2.089	*q* < 0.001
coagulation	2.017	*q* < 0.001
KRAS signaling up	1.996	*q* < 0.001
allograft rejection	1.910	*q* < 0.001
complement	1.762	0.002
IL2/STAT5 signaling	1.702	0.004
IL6/JAK/STAT3 signaling	1.696	0.004
interferon-gamma response	1.687	0.004
UV response dn	1.585	0.013
TNF-alpha signaling via NFkb	1.550	0.019
apical junction	1.464	0.048

## Data Availability

Our expression data were deposited in the GEO database (accession number: GSE172120).

## Supplementary Materials

The Supplementary Materials are available online at https://www.mdpi.com/article/10.3390/ijms22189947/s1.
