# Supplementary material for: Impact of Oncogenic Targets by Tumor-Suppressive miR-139-5p and miR-139-3p Regulation in Head and Neck Squamous Cell Carcinoma
Source: Int J Mol Sci. 2021 Sep 14;22(18):9947. doi: 10.3390/ijms22189947 (PMC8469660; doi:10.3390/ijms22189947)
Supplement: Supplementary file 1 [file ijms-22-09947-s001.zip › 0908Supplemental figures Oncogenic targets by miR-139 regulation in HNSCC Figures .pptx]

## Slide 1
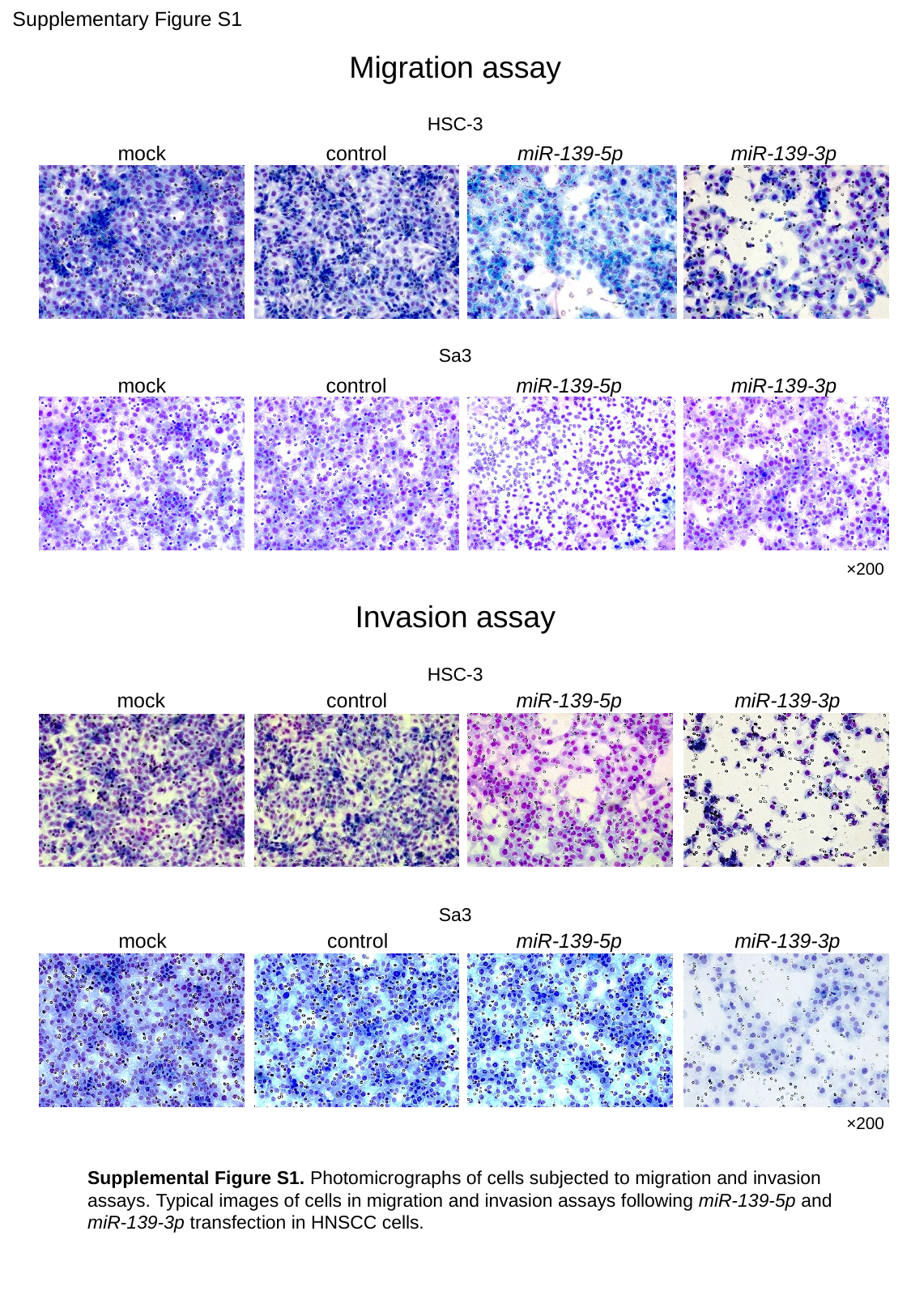

Supplementary Figure S1
Migration assay
HSC-3
control
miR-139-5p
mock
miR-139-3p
Sa3
mock
control
miR-139-5p
miR-139-3p
×200
Invasion assay
HSC-3
mock
control
miR-139-5p
miR-139-3p
Sa3
mock
control
miR-139-5p
miR-139-3p
×200
Supplemental Figure S1. Photomicrographs of cells subjected to migration and invasion assays. Typical images of cells in migration and invasion assays following miR-139-5p and miR-139-3p transfection in HNSCC cells.

## Slide 2
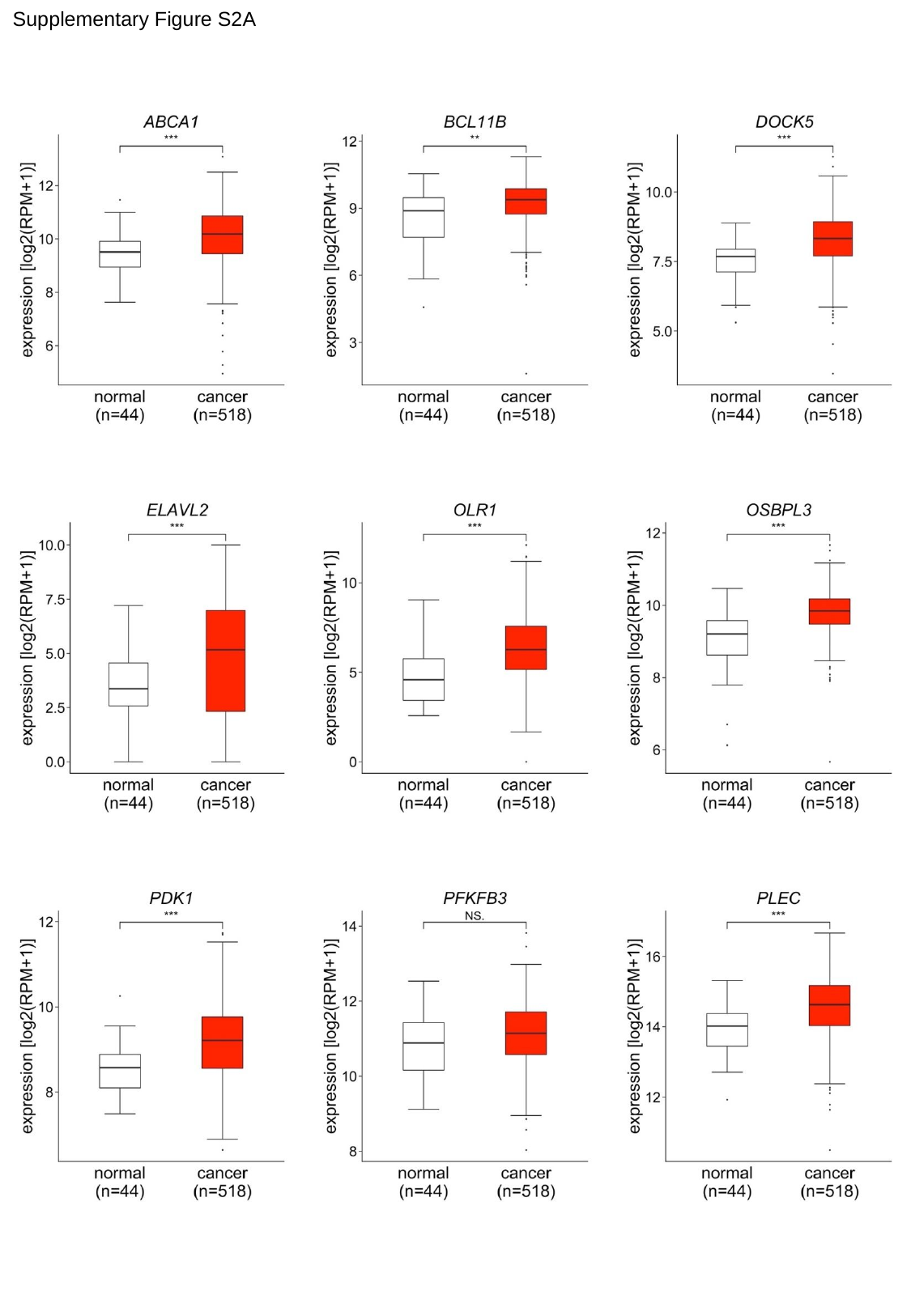

Supplementary Figure S2A

## Slide 3
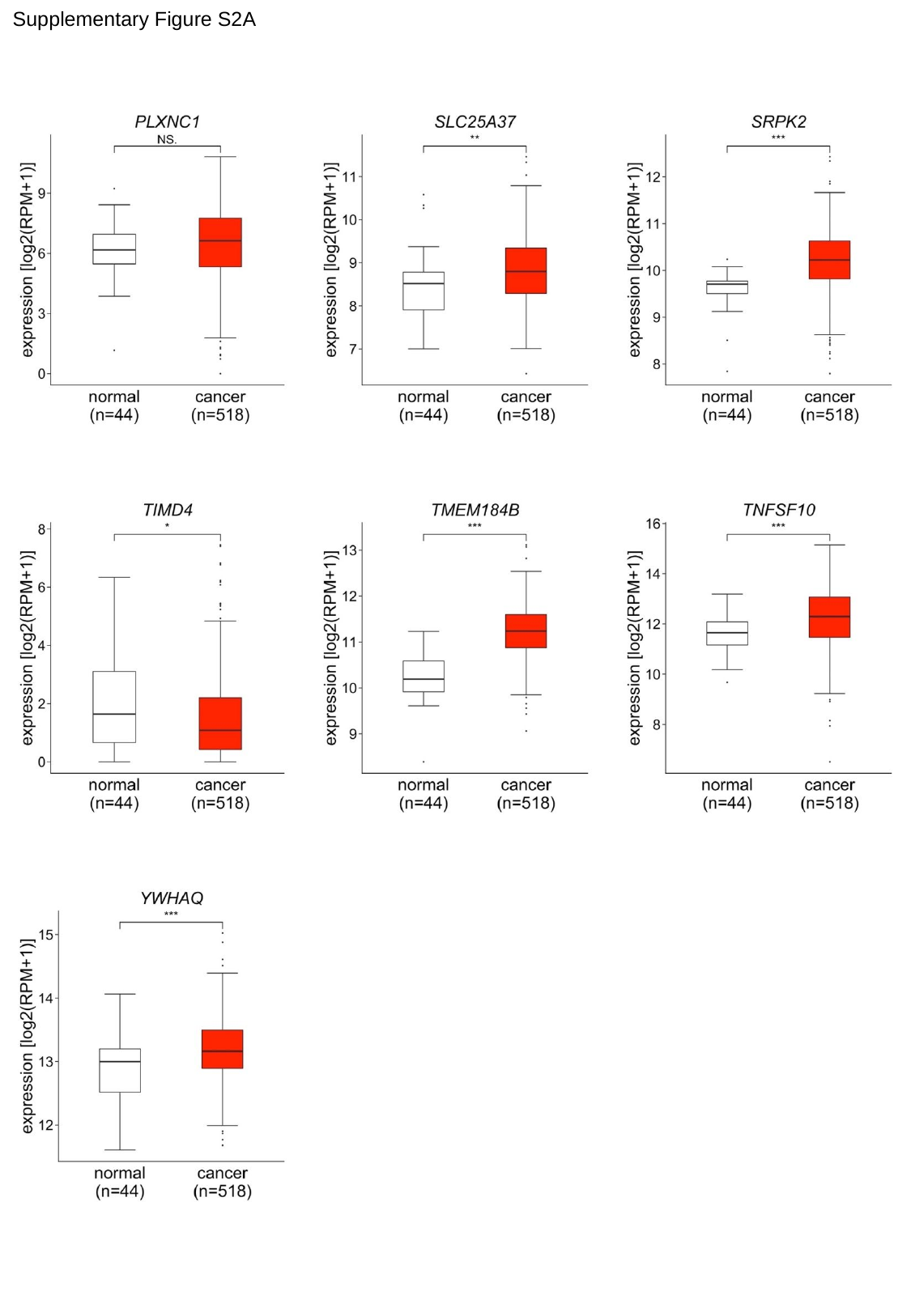

Supplementary Figure S2A

## Slide 4
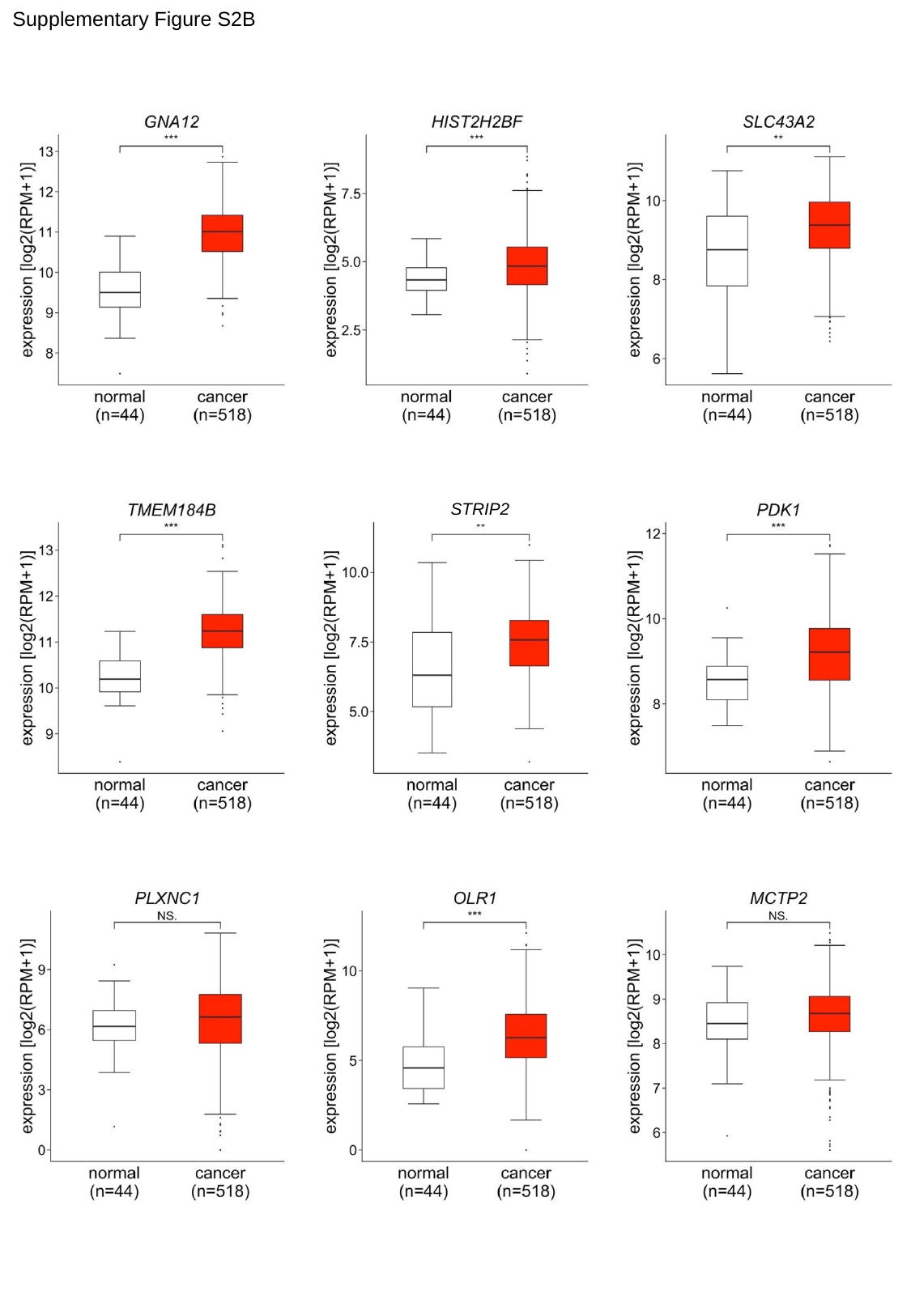

Supplementary Figure S2B
STRIP2

## Slide 5
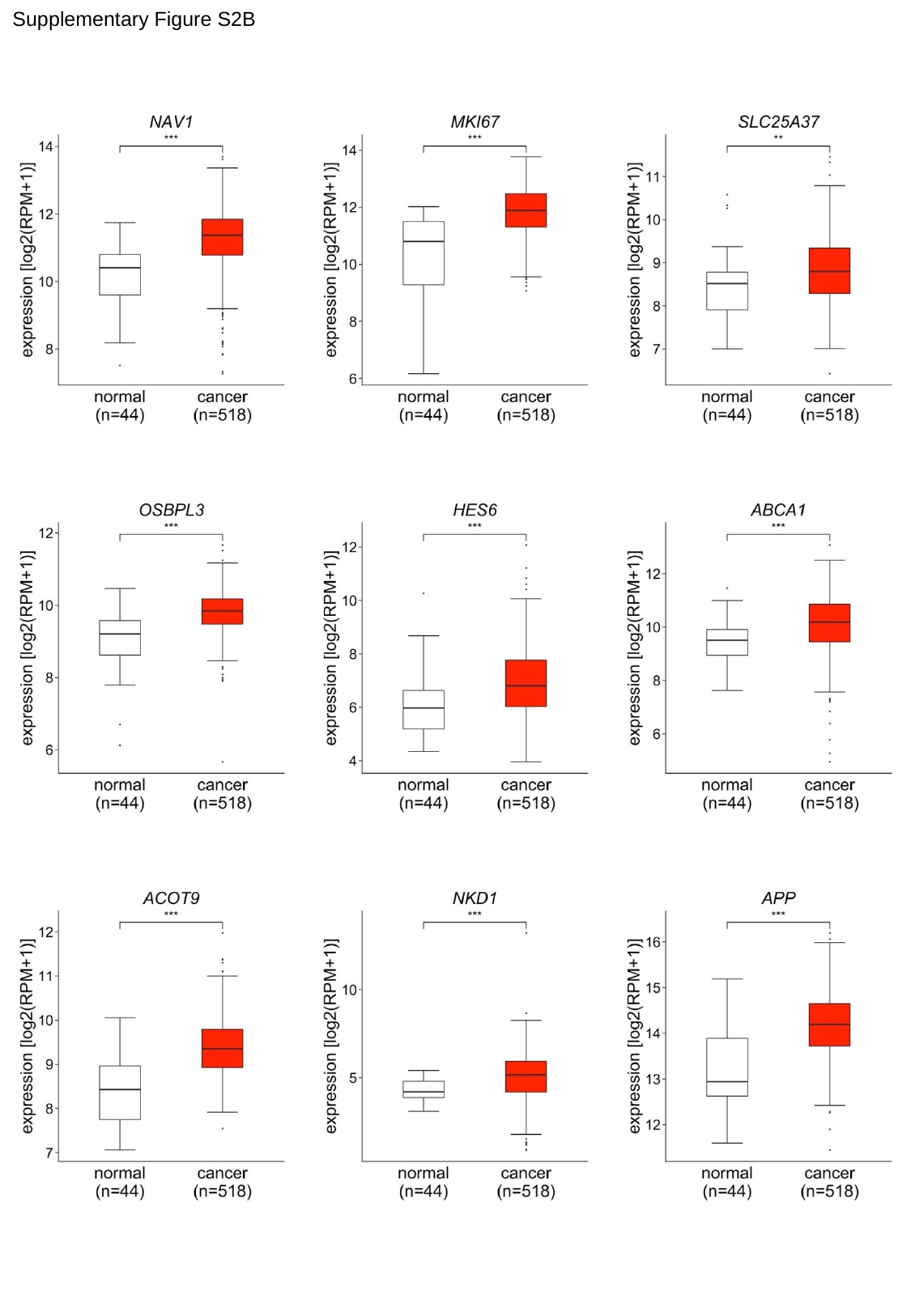

Supplementary Figure S2B

## Slide 6
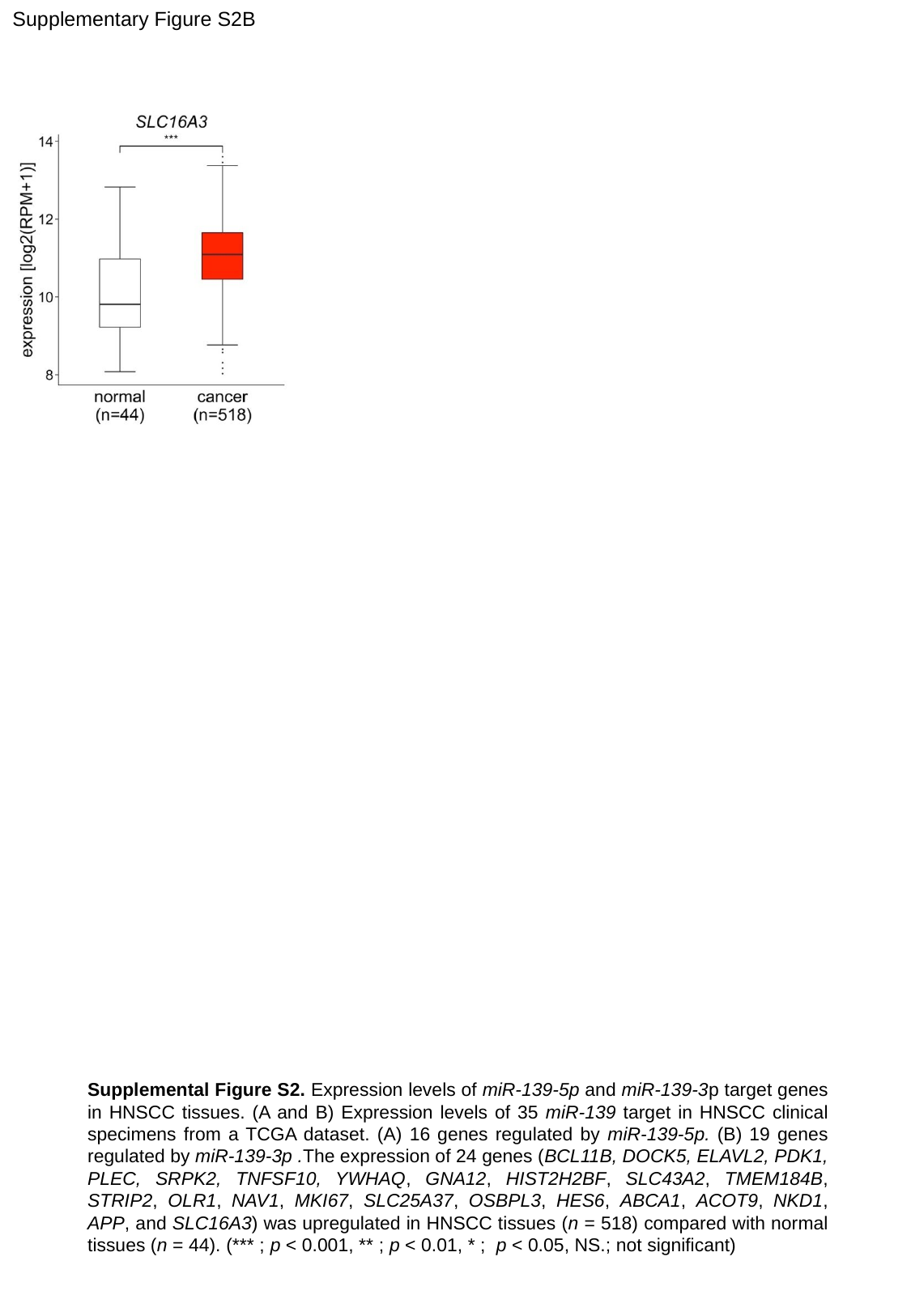

Supplementary Figure S2B
Supplemental Figure S2. Expression levels of miR-139-5p and miR-139-3p target genes in HNSCC tissues. (A and B) Expression levels of 35 miR-139 target in HNSCC clinical specimens from a TCGA dataset. (A) 16 genes regulated by miR-139-5p. (B) 19 genes regulated by miR-139-3p .The expression of 24 genes (BCL11B, DOCK5, ELAVL2, PDK1, PLEC, SRPK2, TNFSF10, YWHAQ, GNA12, HIST2H2BF, SLC43A2, TMEM184B, STRIP2, OLR1, NAV1, MKI67, SLC25A37, OSBPL3, HES6, ABCA1, ACOT9, NKD1, APP, and SLC16A3) was upregulated in HNSCC tissues (n = 518) compared with normal tissues (n = 44). (*** ; p < 0.001, ** ; p < 0.01, * ; p < 0.05, NS.; not significant)

## Slide 7
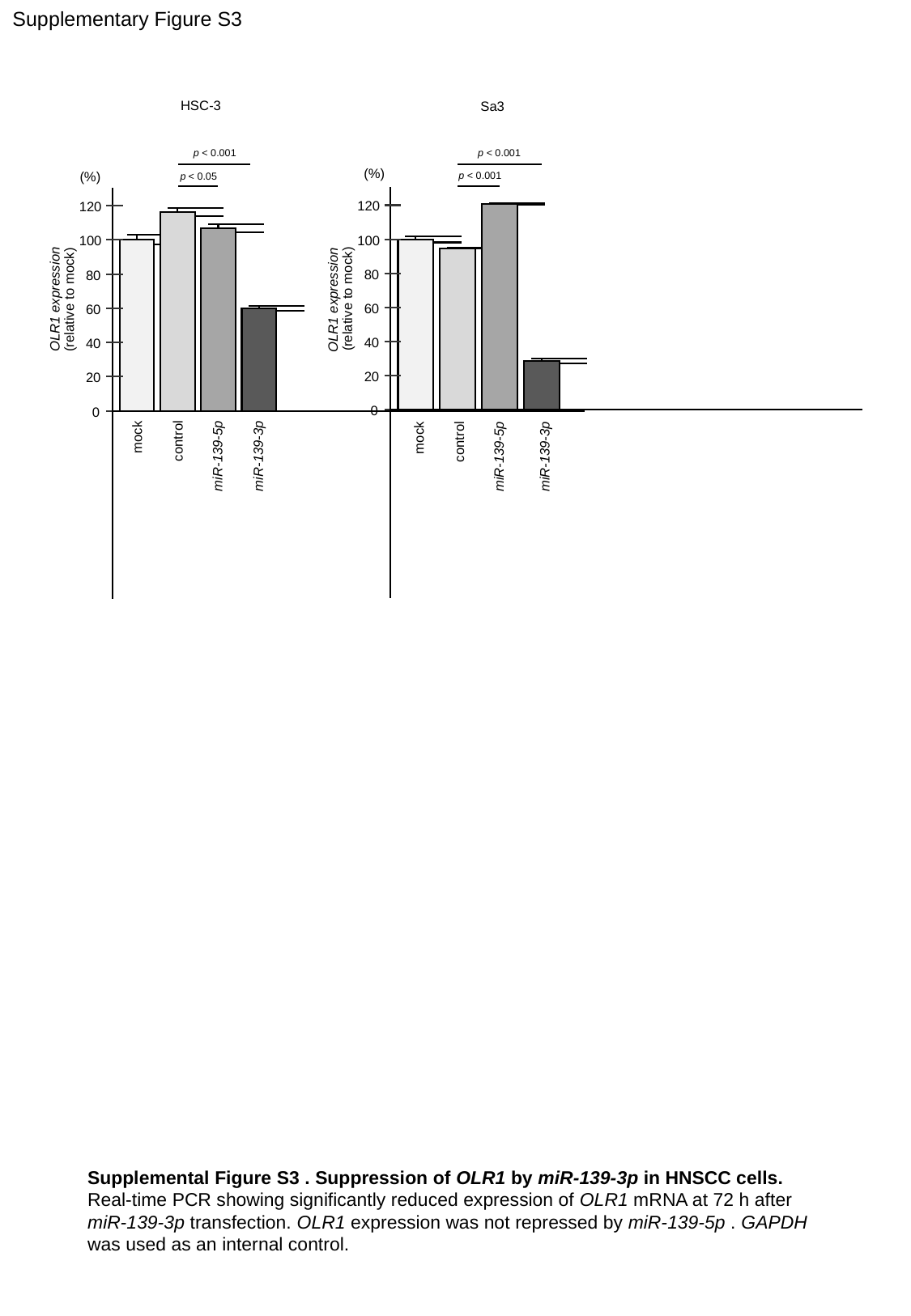

Supplementary Figure S3
HSC-3
Sa3
p < 0.001
p < 0.001
(%)
(%)
p < 0.001
p < 0.05
120
120
100
100
80
80
(relative to mock)
OLR1 expression
(relative to mock)
 OLR1 expression
60
60
40
40
20
20
0
0
mock
mock
control
control
miR-139-5p
miR-139-3p
miR-139-3p
miR-139-5p
Supplemental Figure S3 . Suppression of OLR1 by miR-139-3p in HNSCC cells. Real-time PCR showing significantly reduced expression of OLR1 mRNA at 72 h after miR-139-3p transfection. OLR1 expression was not repressed by miR-139-5p . GAPDH was used as an internal control.

## Slide 8
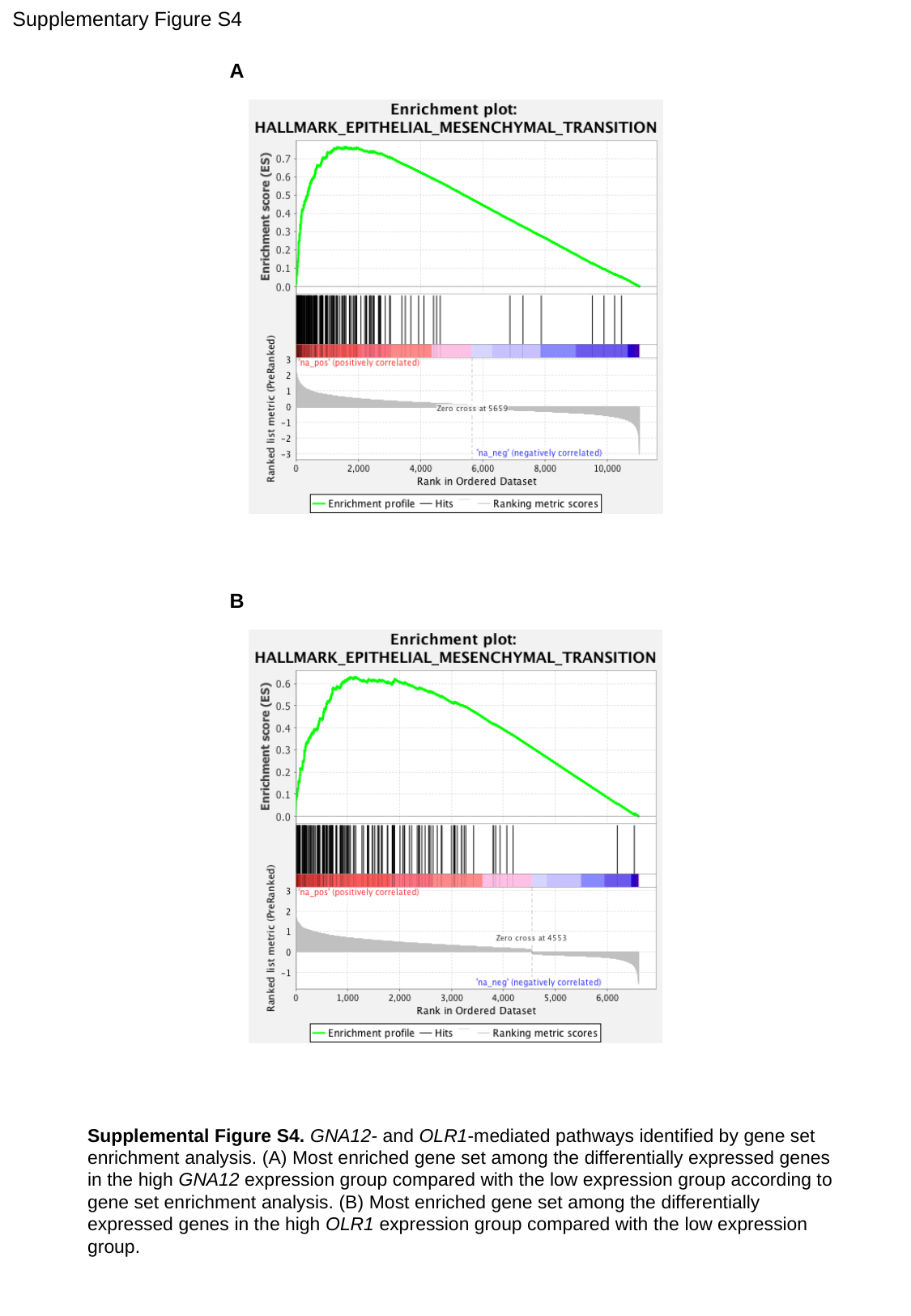

Supplementary Figure S4
A
B
Supplemental Figure S4. GNA12- and OLR1-mediated pathways identified by gene set enrichment analysis. (A) Most enriched gene set among the differentially expressed genes in the high GNA12 expression group compared with the low expression group according to gene set enrichment analysis. (B) Most enriched gene set among the differentially expressed genes in the high OLR1 expression group compared with the low expression group.
